# Characterization and Action Mechanism Analysis of VvmiR156b/c/d-VvSPL9 Module Responding to Multiple-Hormone Signals in the Modulation of Grape Berry Color Formation

**DOI:** 10.3390/foods10040896

**Published:** 2021-04-19

**Authors:** Ziwen Su, Xicheng Wang, Xuxian Xuan, Zilu Sheng, Haoran Jia, Naseri Emal, Zhongjie Liu, Ting Zheng, Chen Wang, Jinggui Fang

**Affiliations:** 1 College of Horticulture, Nanjing Agricultural University, Nanjing 210095, China; 20188042071@njau.edu.cn (Z.S.); 2019804271@njau.edu.cn (X.X.); 2019104029@njau.edu.cn (Z.S.); 2018104010@njau.edu.cn (H.J.); 2019804199@njau.edu.cn (N.E.); 2017204015@njau.edu.cn (Z.L.); zhengting@njau.edu.cn (T.Z.); 15514123@njau.edu.cn (J.F.); 2 Institute of Pomology, Jiangsu Academy of Agricultural Science, Nanjing 210014, China; 2020204007@njau.edu.cn

**Keywords:** VvmiR156b/c/d-*VvSPL9*, hormone signal, anthocyanin, grape

## Abstract

In recent years, more and more reports have shown that the miR156-*SPL* module can participate in the regulation of anthocyanin synthesis in plants. However, little is known about how this module responds to hormonal signals manipulating this process in grapes. In this study, exogenous GA, ABA, MeJA, and NAA were used to treat the ‘Wink’ grape berries before color conversion, anthocyanin and other related quality physiological indexes (such as sugar, aroma) were determined, and spatio-temporal expression patterns of related genes were analyzed. The results showed that the expression levels of VvmiR156b/c/d showed a gradually rising trend with the ripening and color formation of grape berries, and the highest expression levels were detected at day 28 after treatment, while the expression level of *VvSPL9* exhibited an opposite trend as a whole, which further verifies that VvmiR156b/c/d can negatively regulate *VvSPL9*. Besides, VvmiR156b/c/d was positively correlated with anthocyanin content and related genes levels, while the expression pattern of *VvSPL9* showed a negative correlation. Analysis of promoter cis-elements and GUS staining showed that VvmiR156b/c/d contained a large number of hormone response cis-elements (ABA, GA, SA, MeJA, and NAA) and were involved in hormone regulation. Exogenous ABA and MeJA treatments significantly upregulated the expression levels of VvmiR156b/c/d and anthocyanin structural genes in the early stage of color conversion and made grape berries quickly colored. Interestingly, GA treatment downregulated the expression levels of VvmiR156b/c/d and anthocyanin structural genes in the early color-change period, but significantly upregulated in the middle color-change and ripening stages, therefore GA mainly modulated grape berry coloring in the middle- and late-ripening stages. Furthermore, NAA treatment downregulated the expression levels of VvmiR156b/c/d and anthocyanin structural genes and delayed the peak expression of genes. Meanwhile, to further recognize the potential functions of VvmiR156b/c/d, the mature tomato transient trangenetic system was utilized in this work. Results showed that transient overexpression of VvmiR156b/c/d in tomato promoted fruit coloring and overexpression of *VvSPL9* inhibited fruit coloration. Finally, a regulatory network of the VvmiR156b/c/d-VvSPL9 module responsive to hormones modulating anthocyanin synthesis was developed. In conclusion, VvmiR156b/c/d-mediated *VvSPL9* participated in the formation of grape color in response to multi-hormone signals.

## 1. Introduction

Grape berry ripening is an extremely complex physiological and biochemical process, accompanied by the formation of fruit color, flavor, and aroma. Among them, the color of grape berry is an important indicator of fruit quality. As grapes color, sugar and aroma accumulate in the berries. Therefore, the berry color and its related qualities above-mentioned directly affect its price and market competitiveness. Anthocyanin is the main pigment in grapes including cyanidin, delphinidin, peonidin, petunidin, and malvidin. Fruit color development is a very complex metabolic process, which is not only affected by genotype, climatic conditions, and cultivation techniques, but also by plant hormones. In the process of fruit coloring, plant hormones are key regulators and play important roles. A variety of hormones interact to co-regulate the formation of colors such as abscisic acid (ABA), gibberellin (GA), ethylene (ETH), auxin (AUX), and jasmonic acid (JA). The application of exogenous hormones can cause changes in endogenous hormones modulating the color formation. For example, ABA, ETH, and JA treatments promote fruit coloring and ripening; conversely, AUX applications inhibit the corresponding processes [1,2,3,4]. Therefore, the effect and regulation mechanism of plant hormones on plant color has always been a hotspot of research.

MicroRNAs (miRNAs), widely distributed in plants, are very important non-coding RNA regulators with a length of 19–24 nucleotides (nt). They play critical roles in plant growth and development, signal transduction, and the response to stress, mainly via their action in causing either complementarity-dependent cleavage or translational inhibition of the target mRNA molecules at the post-transcriptional level [5,6]. In recent years, more and more reports have shown that miRNAs and their target genes are involved in the regulation of anthocyanin synthesis in plants. In plants such as *Arabidopsis thaliana*, tomato, apple, and grape, miR828 regulates the accumulation of anthocyanin and flavonol by targeting MYB transcription factors such as *PAP1/MYB75*, *PAP2/MYB90*, and *MYB113* [7,8,9,10,11,12,13]; similarly, miR778 and miR827 also regulate these processes through the action of target gene *NLA* [14,15]; and miR858 and target gene *MYB12* are also involved in the regulation of anthocyanin biosynthesis [16,17,18]. Furthermore, multiple studies have reported that miR156 participates in the modulation of anthocyanin synthesis, which has been a hot topic for many researchers on anthocyanin synthesis [19,20,21]. In *Arabidopsis thaliana*, miR156 regulates anthocyanin synthesis by negatively regulating target gene *SPL* through interacting with the *PAP1* protein; *SPL9* affects the transcriptional activation activity of the MYB-BHLH-WD40 complex, which inhibits the expression of anthocyanin synthesis *ANS* and *DFR genes*, and thus decreases the accumulation of anthocyanin [22]. Under stress conditions, the biosynthesis of anthocyanin increases, and miR156-*SPL9* may directly manipulate anthocyanin biosynthesis through *PAP1* and *DFR*, while plants can mediate anthocyanin metabolism through miR156-*SPLs*-*DFR* pathways [23]. In litchi (*Litchi chinensis* Sonn.), miR156a is inversely correlated with the expression profiles of its target genes *LcSPL1/2* and *LcCHI*, and the increased expression of miR156a decreases the transcription level of *LCSPL1*, which downregulates anthocyanin biosynthesis via interaction with *LcMYB1* [24]. All these findings demonstrate that miR156s and their targets are involved in the modulation of color formation. However, up until now, little is known about how miR156s responds to hormones to mediate their targets involving the modulation of color formation.

In our previous study, we confirmed that VvSPL9 is the target gene of VvmiR156b/c/d, which is involved in the regulation of grape berry development and maturation; and miR156s could be in response to gibberellin [25,26,27]. These imply that VvmiR156s-mediated *VvSPL* might respond to the hormone to manipulate grape berry and ripening. However, it is not clear whether the mechanism of miR156-*SPL* response hormone signals regulate grape color formation. To elucidate this, we treated ‘Wink’ (*Vitis vinifera* L.) with exogenous GA, ABA, MeJA, and NAA before the color transition period, and measured the physiological indicators associated with quality (such as sugar, anthocyanins, volatile compound). Then, based on the report of miR156-SPL [22,23,26,27], we verified the hormone-related elements in VvmiR156b/c/d promoter by GUS staining using tobacco transient transformation system. Moreover, qRT-PCR technology was used to detect the spatio-temporal expression characteristics of the VvmiR156b/c/d-VvSPL9 module during fruit coloring, and their modes responsive to hormones under different hormone treatments. Furthermore, the function of VvmiR156b/c/d was determined by their transient overexpression in tomatoes. Finally, this study provides a new research view for the hormone-mediated miRNA-target gene module involved in the regulatory mechanism of anthocyanins biosynthesis, which is conducive to the use of chemical regulation in production to promote fruit coloring.

## 2. Materials and Methods

### 2.1. Plant Materials and Hormone Treatments

The experiment was conducted at Jiangsu Academy of Agricultural Sciences, Nanjing, China from July 2019 to August 2019. A 6-year-old grapevine cv. ‘Wink’ (*Vitis vinifera* L.) were used as the plant material. Based on the results of the preliminary experiments and horticultural practices, grape berry clusters were treated by using 50 mg/L gibberellin (GA), 100 mg/L abscisic acid (ABA), 100 mg/L methyl jasmonate (MeJA), 200 mg/L naphthalene acetic acid (NAA), and 0.01% Tween-80 solution (CK), respectively, one week before color conversion (16 July). Berries were sprayed to a completely wet state and samples were collected at 0 days after treatment (0DAT, 16 July), seven days after treatment (7DAT, 23 July), 14 days after treatment (14DAT, 30 July), 21 days after treatment (21DAT, 6 August), 28 days after treatment (28DAT, 13 August), 35 days after treatment (35DAT, 20 August). Ten clusters were randomly selected from each group of the control and treated plants, and three berry grains were randomly selected from the top, middle, and lower parts of each panicle. After weighing, berries were separated into skin, flesh, and seed, then immediately frozen in liquid nitrogen and stored at −80 °C until use.

In addition, the young leaves were sprayed with 100 μM gibberellin (GA), abscisic acid (ABA), methyl jasmonate (MeJA), naphthalene acetic acid (NAA), and 0.1% Tween-80 Solution (CK). Collected leaves were sampled at 1 h, 3 h, 5 h, and 7 h after treatment and five leaves were repeated for each treatment period. Then, the samples of leaves were immediately placed into liquid nitrogen.

### 2.2. Ultra-Performance Liquid Chromatography (UPLC) Analysis

A sample of 0.5 g of grape skin freeze-dried powder (ground with liquid nitrogen) was accurately weighed into a 15 mL Eppendorf tube, and 10 mL of 0.1% methanolic hydrochloric acid solution was added, and placed in a refrigerator at 4 °C for 24 h in the dark. Then, the supernatant was collected and filtered with a 0.45 μm nylon filter. Extracts were analyzed by ultra-performance liquid chromatography (UPLC, Waters, Milford, OH, USA). Separation was achieved with a Zorbax SB-C18 column (50 × 3.0 mm, 1.8 μm), and a binary solvent system of (A) 1% aqueous formic acid (*v/v*) and (B) acetonitrile. The gradient for buffer B was 5% for 2 min, 5~95% for 15 min, and 95% for 2 min. Anthocyanin were monitored with a flow rate of 1.00 mL/min, a column temperature of 25 °C, and a detection wavelength of 530 nm. The chromatographic peaks were identified by comparison with authentic standards of cyanidin-3-O-glucoside chloride (Figure S1).

### 2.3. Gas Chromatography-Mass Spectrometer (GC-MS) Analysis

After freeze-thawing the samples, each replicate sample was pitted and crushed under liquid nitrogen. For each extraction, we weighed 3 g of grape flesh, 3 g NaCl (promotes ionization), and 2 μL octanal ethanolic solution (81.8 μg/μL, added as an internal standard) and placed them into a 20 mL clean capped vial. [28,29]. A 50/30 μm polydimethylsiloxane/divinylbenzene/carboxen (PDMS/DVB/CAR) solid-phase microextraction (SPME) extraction head (Supelco, Bellefonte, PA, USA) was inserted for GC-MS determination. VOC (volatile organic compound) analysis (Figure S2) was performed on a GC-MS/MS Quantum TSQ 9000/TRACE 1310 (Thermo Fisher Scientific, USA) using a J&W 122-4732 DB-17ms (30 m, 0.25 mm, 0.25 μm) column. The temperature ramp was: 40 °C for 5 min; then 2 °C/min to 70 °C for 2min; 3 °C/min to 120 °C, then 5 °C/min to 150 °C/min, and finally to 10 °C/min raised to 220 °C for 2 min. The transfer line temperature was 280 °C and the MS conditions were as follows: the mass spectrometer adopted the EI mode, voltage was 70 eV; ion source temperature was 230 °C; scanning rate was 2.88 scan/s; mass spectrometry detection range was 29–540 *m/z*, the carrier gas was helium, and the flow rate was 1.0 mL/min.

### 2.4. Total RNA and DNA Isolation, cDNA Synthesis

The total RNA and DNA were isolated from 200 mg of the grapevine plant materials using the modified cetyltrimethylammonium bromide (CTAB) method. The concentration of RNA and DNA samples were measured by a UV-1800 spectrophotometer and the quality and quantity of RNA and DNA samples were assessed by 1.5% agarose gel electrophoresis. The cDNA was synthesized from total RNA using the PrimeScriptTM^RT^ Reagent Kit (Takara, Japan) and stored at −40 °C following the manufacturer’s instructions.

### 2.5. Cis-Element Analysis of The Promoters from VvMIR156b/c/d

We obtained promoter sequences (approximately 2000 bp upstream of genes) of VvMIR156b/c/d from the Grape Genome Browse (http://www.genoscope.cns.fr/externe/GenomeBrowser/Vitis/, accessed on 14 April 2021). Promoter cis-elements were further analyzed by PlantCARE software (http://bioinformatics.psb.ugent.be/webtools/plantcare/html/, accessed on 14 April 2021), which provided a basis for exploring the biological functions of VvMIR156b/c/d.

### 2.6. Construction of the VvMIR156b/c/d Promoter Vector and Agrobacterium-Mediated Tobacco Transient Transformation

According to the VvmiR156b/c/d promoter sequences (Table S2), the upstream and downstream primers containing BamHⅠ and HindⅢ restriction sites were designed [30]. To develop pBI121-VvmiR156b/c/d-GUS constructs, 2000 bp promoter regions of VvmiR156b/c/d were independently cloned and fused with the GUS reporter gene to replace the pBI121-35SCaMV promoter in accordance with the instructions of the FastPure Gel DNA Extraction Mini Kit (Vazyme, Nanjing, China). The recombinant plasmid vectors were transferred into Agrobacterium EHA105, and positive clones were identified through PCR. Then, a single colony was cultured in LB liquid medium supplied with rifampin (100 μg/mL) and kanamycin (100 μg/mL). After OD600 = 0.5, the bacteria were pelleted and resuspended in suspension buffer (10 mM MgCl_2_, 10 mM MES, and 150 μM AS) and left to stand at room temperature for 3~4 h.

Six-week-old wild tobacco (*Nicotiana benthamiana*) leaves were selected for Agrobacterium tumefaciens infection, and the transient expression of the target protein in tobacco was carried out. The injected tobacco was kept in the dark for two days and the leaves were treated with 100 μM GA, ABA, MeJA, and NAA. On day 3, leaves were collected and each treatment was repeated with five leaves and the experiment was repeated three times.

### 2.7. β-Glucuronidase (GUS) Staining and Activity Detection

The β-glucuronidase (GUS) gene is a commonly used reporter gene, which could reflect promoter expression levels. GUS histochemical staining was performed on tobacco leaves using the GUS Staining Kit (Solarbio, Beijing, China). First, tobacco leaves were punched and soaked in GUS staining solution. The vacuum pump was used for 30 min under a 0.085 kPa condition, then stained overnight at 37 °C. Finally, a 75% ethanol solution was used to decolorize. GUS expression was observed and photographed under a microscope (Leica M205FA, Wetzlar, Germany). Moreover, the quantification of GUS activity was determined using the fluorometric 4-methylumbelliferyl-b-D-glucuronide (MUG) method. One unit of GUS activity was defined as 1 nM of 4-methylumbelliferon (4-MU) generated per milligram of soluble protein per minute. Three leaves were infiltrated for each construct in each independent experiment and then combined to detect GUS activity.

### 2.8. Construction of vvmiR156b/c/d and VvSPL9 Overexpression Vectors and Instantaneous Injection of Tomato

Based on the precursor sequences of VvmiR156b/c/d downloaded from the miRBase database (http://www.mirbase.org/, accessed on 14 April 2021), specific primers containing NcolⅠ and SpeⅠ restriction sites were designed for PCR amplification (Table S1). PCR amplification was conducted as follows: 5 min at 94 °C, 30 s at 94 °C, 30 s at 56 °C, 1 min at 72 °C, 35 cycles, and extension at 72 °C for 10 min. First, the PCR fragments were purified and ligated into the vector pMD19-T and transformed into DH5α competent cells. Second, the target fragment and pCAMBIA-1302 vector were digested with NcolⅠ and SpeⅠ and ligated with the T4 DNA ligase, then transformed into DH5α competent cells. Finally, the combined plasmid vectors were transferred into *Agrobacterium* EHA105, and positive clones were identified through PCR.

In transgenic research, grape transgenic technology has not yet been perfected and matured, while tomato is a model plant and has formed a series of mature technical systems. Therefore, instant injection of tomato can be used to quickly verify the function of genes. Each *Agrobacterium* strain of VvmiR156b/c/d and *VvSPL9* was cultured in LB liquid medium supplied with rifampin (100 μg/mL) and kanamycin (100 μg/mL). After OD600 = 0.8, the bacteria were pelleted and resuspended in suspension buffer (10 mM MgCl_2_, 10 mM MES, and 150 μM AS) and left to stand at room temperature for 3~4 h. Finally, a syringe was used to inject the *Agrobacterium* liquid into the tomato before the color change [31]. Ten uniformly sized fruit were used in the infiltration experiment, and the experiment was repeated three times. The pCAMBIA1302 vector universal primer was used for PCR amplification to obtain striped positive plants.

### 2.9. Gene Expression Analysis

According to the principle of fluorescent quantitative PCR primer design, qRT-PCR primers (Tables S3–S5) were designed on Primer3 (http://bioinfo.ut.ee/primer3-0.4.0/primer3/, accessed on 14 April 2021). Using cDNA as a template, *VvActin* (Accession number: XM_002273532) was used as a reference gene for qRT-PCR. qRT-PCR was conducted following the instructions of the SYBR Premix Ex Tap^TM^ Kit (TaKaRa, Dalian, China). The amplification system was 10 μL: cDNA 1 μL, upstream and downstream primer 0.3 μL each, TransStart^®^ Tip Green qPCR SuperMix 5 μL, dd H_2_O 3.4 μL. The reaction procedure was 95 °C for 300 s, followed by 40 cycles at 95 °C for 10 s and 60 °C for 30 s. The relative expression level was calculated with the formula 2^−ΔΔCT^ = normalized expression ratio. Each PCR assay was carried out by three biological replicates, and each replicate corresponded to three repeats of separate experiments.

### 2.10. Statistical Analysis

Data processing, difference significance analysis, and correlation analysis were done using Excel and SPSS 16.0. Asterisks indicated statistically significant differences at (* *p* < 0.05; ** *p* < 0.01) as determined by the Student’s *t*-test.

## 3. Results

### 3.1. The Effect of Different Hormones on the Color and Quality Traits of Grape Berry

#### 3.1.1. Effects of Different Hormones on Grape Phenotypic Characteristics

**Figure 1:**
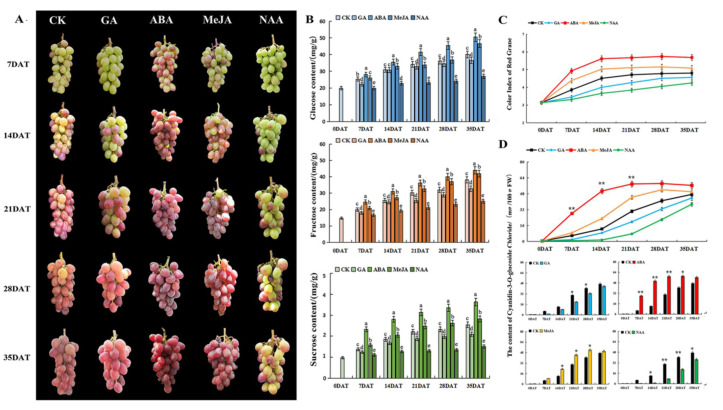
Physiological characteristics of ‘Wink’ grape treated with different hormones. (**A**) Morphological and color change. (**B**) The contents of soluble sugars (glucose, fructose, and sucrose). (**C**) Color Index of Red Grape (CIRG). (**D**) The contents of anthocyanins (cyanidin-3-O-glucoside chloride). Berry samples were randomly collected at 0 days after treatment (0DAT, 16 July), seven days after treatment (7DAT, 23 July), 14 days after treatment (14DAT, 30 July), 21 days after treatment (21DAT, 6 August), 28 days after treatment (28DAT, 13 August), and 35 days after treatment (35DAT, 20 August). Different letters on the bar graph indicate significant differences, and the same indicates no significant differences (*p* < 0.05).

As showed in Figure 1, ABA and MeJA were beneficial to the color conversion and maturation of grapes, while GA and NAA treatments were not conducive to the color conversion and maturation of grape berries. In the early stages of color-change, the fruit growth rate was faster. As the berries tend to mature, the growth rate of berries gradually slows down and tends to flatten as a whole. Moreover, ABA and MeJA treatments increased the size and weight of berries, while GA and NAA treatments inhibited the expansion of the berries (Table 1).

#### 3.1.2. Effects of Different Hormones on Grape Sugar Content

The sugars in grapes mainly include glucose, fructose, and sucrose, in which the contents of glucose are the highest, and the proportion of sucrose is the smallest (Figure 1B). With the growth and development of fruit, the contents of soluble solids and three kinds of soluble sugar gradually increased, and the contents reached the highest at the ripening period. After treatments of ABA and MeJA on grape berries, the contents of soluble solids and soluble sugars (glucose, fructose, and sucrose) increased significantly. In contrast, after GA and NAA treatment on grape berries, the contents of soluble solids and soluble sugars decreased; in particular, the NAA treatment inhibited berry veraison and ripening.

#### 3.1.3. Effects of Different Hormones on Grape Coloring

To evaluate the appearance and color of grapes more accurately, the color index of red grape (CIRG) was used to evaluate the appearance and color of berries. CIRG range: CIRG < 2 is green, 2 < CIRG < 4 is pink, 4 < CIRG < 5 is red, 5 < CIRG < 6 is purple, CIRG > 6 is blue-black or purple-black. The results of the CIRG value evaluation of fruit appearance and color (Figure 1C) were consistent with the field observation of the sample (Figure 1A). To analyze the effects of different hormone treatments on anthocyanins in grape pericarp, contents of cyanidin-3-O-glucoside chloride were detected by UPLC (Figure S1). It can be observed from Figure 1D that the contents of cyanidin-3-O-glucoside chloride after ABA and MeJA treatments increased significantly, while its contents decreased after GA and NAA treatments. Interestingly, the contents were not always increased. After ABA treatment, the contents of cyanidin-3-O-glucoside chloride peaked (58.18 mg/100 g FW) at 21DAT, and then remained basically unchanged. After MeJA treatment, the contents of cyanidin-3-O-glucoside reached the highest peak (51.91 mg/100 g FW) at 28DAT and then decreased slightly. Therefore, ABA and MeJA treatments were beneficial to the accumulation of anthocyanins, while GA and NAA treatment decreased the accumulation of anthocyanins.

#### 3.1.4. Effects of Different Hormones on Grape Volatile Compound

The volatile compound identification and relative contents were based on data including the retention time, BP area, and BP height as obtained from GC-MS (Figure S2 and Table S6). A variety of volatile components were detected in grape flesh, mainly including acids, alcohols, esters, terpenes, aldehydes, ketones, and phenols, among which the contents of alcohol substances were the highest, followed by aldehydes. From the qualitative and quantitative analyses, there were some differences in the types and relative contents of volatile compounds treated with different hormones. In the control samples (CK), 2-hexenal, hexanal, and 3-hexen-1-OL (Z)- accounted for 31.59%, 9.80%, and 9.22% of the total volatiles, respectively. In GA treated samples, 2-hexenal, henzyl 2-chloroethyl sulfone, and cyclopropane, (1-methylethylidene)- accounted for 33.35%, 20.37%, and 12.56% of the total volatiles, respectively, while 2-hexenal, hexanal, and 3-hexen-1-ol accounted for 40.93%, 11.44%, and 10.47% in the ABA treated samples. In the MeJA treated samples, the three highest volatile substances were 2-hexenal, 1, 3, 5-cycloheptatriene, and trans-2-hexenol, accounting for 30.91%, 7.61%, and 5.96% of the total volatile substances respectively, while 2-hexenal, 3-hexen-1-ol, (Z)-, and formamide, N,N-dibutyl- accounted for 9.97%, 6.98%, and 6.70% of total volatiles, respectively, in the NAA treatment samples. Among the five samples, the total amount of volatile compounds in the MeJA treatment samples was the highest, followed by ABA and GA, while the total amount of volatile compounds in the CK and NAA treatment samples were the lowest. Compared with the control group (CK), the total amount of volatile compounds in grape berries treated with MeJA and ABA increased by 81.97% and 28.69%, respectively.

### 3.2. Cis-element Analysis and GUS Activity Detection of VvMIR156b/c/d Promoters in Response to Exogenous Hormones

**Figure 2:**
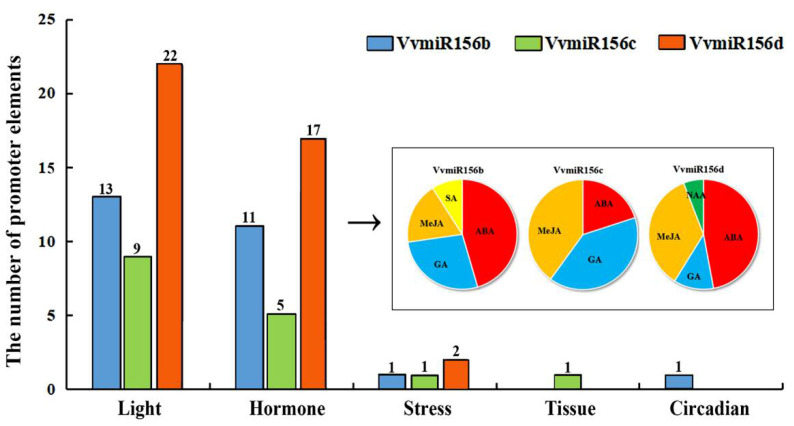
Motif analysis of the VvMIR156b/c/d promoters.

As shown in Figure 2 and Table S7, promoter cis-elements of VvMIR156b/c/d can be classified as light-related, hormone-related, stress-related, tissue-specific, and circadian-related elements based on their potential functions. Among these five groups, the number of light-related elements was the largest, which might be due to the important role of light in photosynthesis for plant growth and development, followed by the hormone-related elements, whereas the number of cis-elements, responsive tissue-specific and circadian-related, were detected to be less. These motifs could indicate the corresponding miRNAs and potential functions of genes.

Hormone-related motifs in their promoter regions were scanned and it was found that almost all VvmiR156b/c/d had similar hormones-related motifs including the ABA, GA, and MeJA responsive motifs (Figure 2). Furthermore, the SA-responsive element (TCA-element) and auxin-responsive element (TGA-element) in VvmiR156d were also found. Among these hormone-related elements, the number of cis-elements responsive to ABA and MeJA was the greatest, followed by the GA and auxin-responsive elements. This indicated that VvmiR156b/c/d might answer hormone signals and be involved in these several plant hormone-related pathways, and jointly participate in the regulation of grape growth and development.

**Figure 3:**
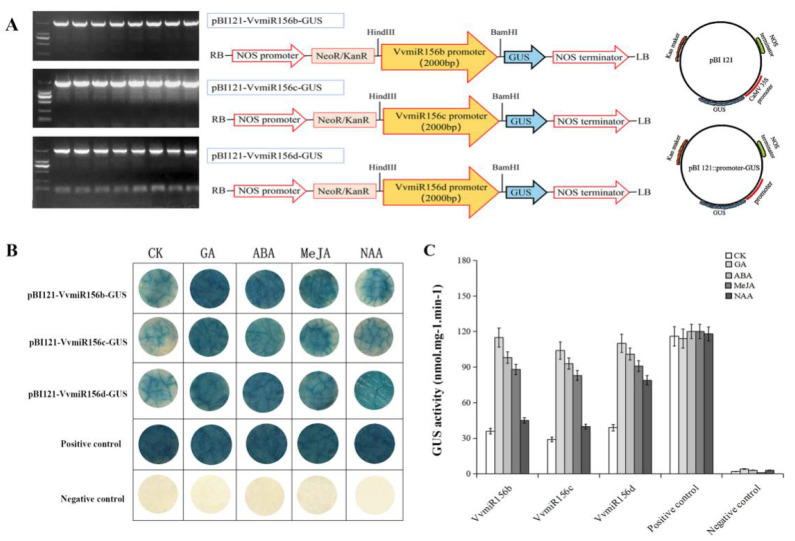
GUS staining analysis of VvMIR156b/c/d promoters in response to exogenous hormones. (**A**) The promoter vector construction. (**B**) GUS staining. (**C**) GUS activity analysis.

The promoter vectors (pBI121-VvmiR156b/c/d-35SCaMV) were conducted as shown in Figure 3A. After GUS histochemical staining, it was found that full-length promoter vectors of VvmiR156b/c/d (pBI121-VvmiR156b/c/d-35SCaMV) were stained blue, and the positive control (pBI121 carrier with 35SCaMV promoter) also appeared blue, while the negative control (pBI121 carrier without 35SCaMV promoter) did not show a blue color, indicating that the promoter of VvmiR156b/c/d (pBI121-VvmiR156b/c/d-35SCaMV) had GUS activity. After GA, ABA, MeJA, and NAA treatments, GUS staining of the VvmiR156b/c/d promoter vectors (pBI121-VvmiR156b/c/d-35SCaMV) deepened and GUS activities were enhanced to a certain extent, while the positive control (pBI121 carrier with 35SCaMV promoter) remained almost unchanged (Figure 3B,C). Our results show that the VvmiR156b/c/d promoter regions contained multi-hormone cis-elements and responded to multi-hormone signals.

### 3.3. The Expression Characteristics of VvmiR156b/c/d and VvSPL9 Responsive to Various Hormones in the Modulation of Grape Color Formation

#### 3.3.1. Short-Term Response of VvmiR156b/c/d and *VvSPL9* to Exogenous Hormones in ‘Wink’ Leaves

**Figure 4:**
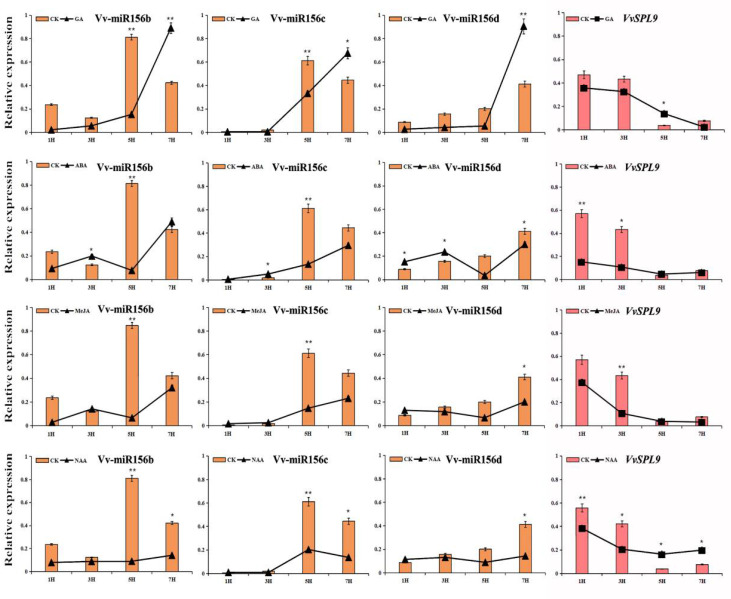
Differential expression of VvmiR156b/c/d and their *VvSPL* target genes in response to different hormone treatments at different time points [1 h after treatment (1HAT), 3HAT, 5HAT, 7HAT] of leaf development. Asterisks indicated statistically significant differences at (* *p* < 0.05; ** *p* < 0.01) as determined.

To identify the transient induction effect of exogenous hormones on VvmiR156b/c/d and *VvSPL9*, the treated grape leaves with GA, ABA, MeJA, and NAA were used to detect the expression of VvmiR156b/c/d and *VvSPL9*. As Figure 4 shows, in the control samples, VvmiR156b/c/d exhibited lower expression levels at 1HAT and 3HAT, while showed higher expression levels at 5HAT and 7HAT; conversely, their targets showed the opposite expression modes, indicating the potential negative regulatory roles of VvmiR156b/c/d on *VvSPL9*. Furthermore, we revealed that among the four hormone treatments, GA significantly upregulated the expressions of VvmiR156b/c/d at 7HAT and decreased *VvSPL9* at the corresponding stage, thereby enhancing their negative correlation at the expression levels, which suggests that GA promoted the negative regulatory roles of VvmiR156b/c/d on *VvSPL9*. Although the other three hormone treatments nearly downregulated the expression levels of VvmiR156b/c/d and VvSPL9 at all times, the expression levels of VvmiR156b/c/d and VvSPL9 under the ABA, MeJA, and Auxin hormones treatments could still maintain a negative correlation, implying that these hormones mediate the negative regulatory roles of VvmiR156b/c/d on VvSPL9. Meanwhile, the results suggest that these modules had a stronger response to GA than the remaining three hormones ABA, MeJA, and Auxin, similar to the GUS stain results above (Figure 3).

#### 3.3.2. Long-Term Response of VvmiR156b/c/d and *VvSPL9* to Exogenous Hormones in ‘Wink’ Berry Skin

**Figure 5:**
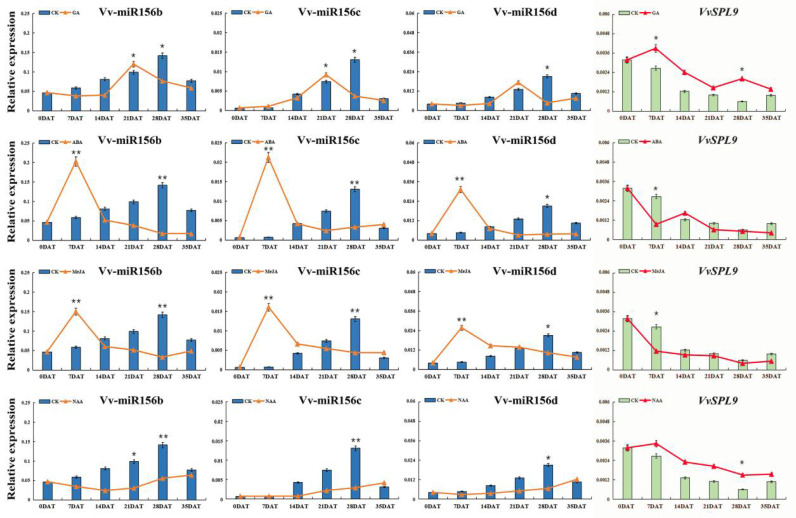
Differential expression of VvmiR156b/c/d and their *VvSPL* target genes in response to different hormone treatments at different time points [seven days after treatment (7DAT), 14DAT, 21DAT, 28DAT, 35DAT] of berry development. Asterisks indicated statistically significant differences at (* *p* < 0.05; ** *p* < 0.01) as determined.

To further identify the long-term expression pattern of VvmiR156b/c/d and *VvSPL9* during grape color conversion, qRT-PCR was used for relative expression levels. As Figure 5 shows, VvmiR156b/c/d had a similar expression pattern, in which VvmiR156b had the highest expression level, followed by VvmiR156d, and VvmiR156c had the lowest expression levels. With the ripening and color transformation of grape berries, VvmiR156b/c/d showed a gradually rising trend, and the highest expression level was detected at 28DAT, while the expression level of *VvSPL9* showed the opposite trend as a whole, which further verified that VvmiR156b/c/d could negatively regulate the expression of *VvSPL9*.

Interestingly, different hormone treatments exhibited three regulatory modes for VvmiR156b/c/d-*VvSPL9*. GA treatment downregulated the expression level of VvmiR156b/c/d in the early color-change period (0DAT–14DAT), upregulated the expression level of VvmiR156b/c/d in the middle color-change period (21DAT), and then downregulated the expression level of VvmiR156b/c/d in the berry mature period (28DAT-35DAT); in contrast, GA treatment increased the expression of *VvSPL9* in the early color-change period (0DAT–14DAT) and berry mature period (28DAT), suggesting that GA treatment significantly enhanced the negative correlation of vmiR156b/c/d-*VvSPL9*, and mainly regulated berry coloring in the middle color-change period. ABA and MeJA treatments significantly increased the expression of VvmiR156b/c/d in the early stage of color conversion (7DAT) and downregulated the expression of VvmiR156b/c/d in the late stage of color conversion (21DAT-35DAT). Furthermore, *VvSPL9* had a stronger regulatory effect in the early stage of color conversion (7DAT), which indicated that ABA and MeJA treatments promoted berry coloring in the early color-change period. Moreover, NAA was a negative regulator of VvmiR156b/c/d. NAA significantly downregulated the expression of VvmiR156b/c/d in berry mature period (28DAT), and NAA showed a gentle upregulated effect on *VvSPL9*. In conclusion, we preliminarily concluded that VvmiR156b/c/d had the earliest response to ABA and MeJA, followed by GA, while NAA had the latest response to VvmiR156b/c/d.

### 3.4. Expression Modes of Genes Related to Anthocyanin Synthesis by Different Hormone Treatments

**Figure 6:**
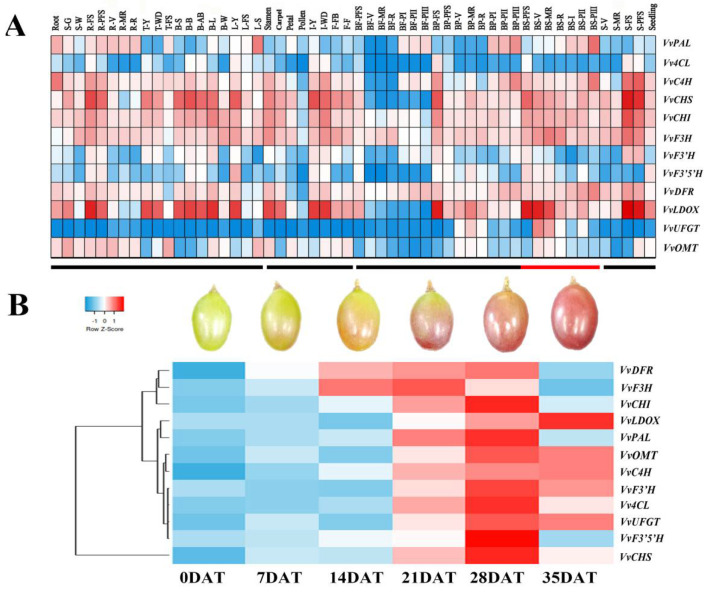
Expression profiles of anthocyanin-related genes. (**A**) The expression of anthocyanin-related genes in different organs and tissues. (**B**) The expression of anthocyanin-related genes of different development periods. Root: root; S-G: green stem; S-W: woody stem; R-FS: rachis fruit set; R-PFS: rachis post fruit set; R-V: rachis veraison; R-MR: rachis mid-ripening; R-R: rachis ripening; T-Y: young tendril; T-WD: well developed tendril; T-FS: mature tendril; B-S: bud swell; B-B: bud burst; B-AB: bud after-burst; B-L: latent bud; B-W: winter bud; L-Y: young leaf; L-FS: mature leaf; L-S: senescencing leaf (pool of leaves at the beginning of leaf fall); Stamen; Carpel: Carpel; Petal: Petal; Pollen: Pollen; I-Y: young inflorescence; I-WD: well developed inflorescence; F-FB: flowering begins; F-F: flowering; BF-PFS: berry flesh post fruit set; BF-V: berry flesh veraison; BF-MR: berry flesh mid-ripening; BF-R: berry flesh ripening; BF-PI: berry flesh post-harvest withering I; BF-PⅡ: berry flesh post-harvest withering Ⅱ; BF-PⅢ: berry flesh post-harvest withering Ⅲ; BP-FS: berry pericarp fruit set; B-PFS: berry pericarp post-fruit; BP-V: berry pericarp veraison; BP-MR: berry pericarp mid-ripening; BP-R: berry pericarp ripening; BP-PI: berry pericarp post-harvest withering I; BP-PⅡ: berry pericarp post-harvest withering Ⅱ; BP-PⅢ: berry pericarp post-harvest withering Ⅲ; BS-PFS: berry skin post fruit set; BS-V: berry skin veraison; BS-MR: berry skin mid-ripening; BS-R: berry skin ripening; BS-PI: berry skin post-harvest withering I; BS-PⅡ: berry skin post-harvest withering Ⅱ; BS-PⅢ: berry skin post-harvest withering Ⅲ; S-V: Seed veraison; S-MR: Seed mid-ripening; S-FS: Seed fruit set; S-PFS: Seed post fruit set; Seeding: Seeding.

The biosynthesis of anthocyanins is controlled by structural and regulatory genes. In order to investigate the temporal and spatial expression patterns of anthocyanin structural genes, we analyzed the expression patterns of 12 anthocyanin structural genes based on the transcriptional mapping data of 54 tissues, organs, and development stages [32]. In the expression profile, the red color indicates strong gene expression, while blue indicates weak gene expression (Figure 6A). *VvC4H*, *VvCHS*, *VvCHI*, *VvF3H*, *VvDFR*, *VvLDOX,* and *VvOMT* were highly expressed in grape roots, stems, buds, and rachis; *VvCHS*, *VvCHI*, *VvF3H,* and *VvLDOX* were also strongly expressed in flower and seed; moreover, *VvPAL*, *VvF3H*, *VvDFR,* and *VvLDOX* were also strongly expressed in the berry, while *VvUFGT* was only specifically highly expressed in the berry skin and pericarp. In addition, we found that in flesh, most of the anthocyanin synthesis-related genes were highly expressed in the berry flesh ripening period. In pericarp, most of the anthocyanin synthesis-related genes were highly expressed in the berry pericarp mid-ripening period. In the peel, most of the anthocyanin synthesis-related genes were highly expressed in the berry skin veraison period.

**Figure 7:**
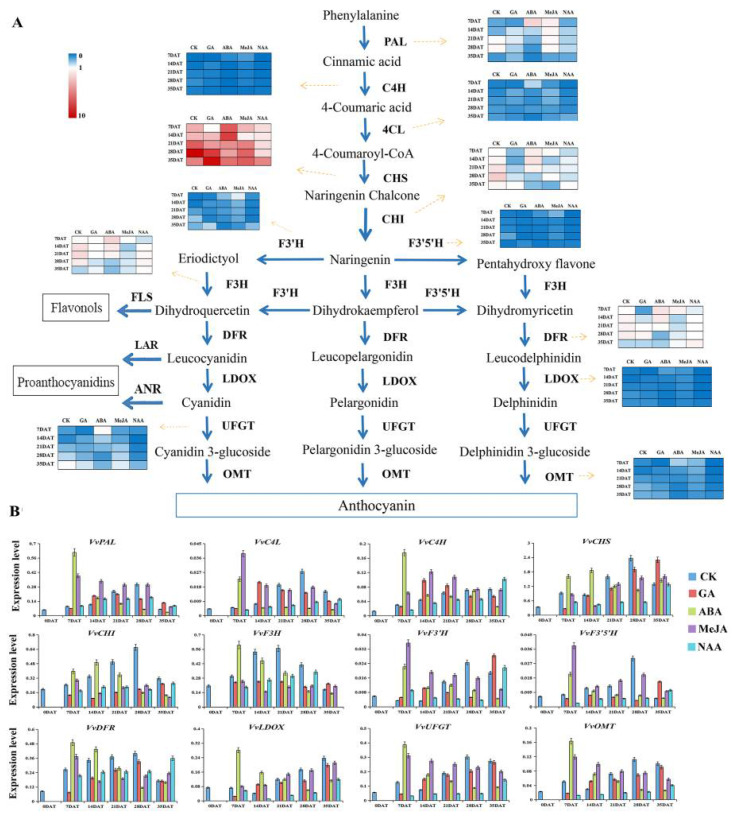
Expression modes of genes related to anthocyanin synthesis by different hormone treatments. (**A**) Anthocyanin metabolism pathway. (**B**) The expression of anthocyanin-related genes by different hormone treatments.

qRT-PCR analysis showed that anthocyanin structural genes had a similar expression trend to VvmiR156b/c/d. With the ripening and color formation of grape berries, the anthocyanin structural genes began to be rapidly upregulated. The expression levels of most anthocyanin structural genes (*VvPAL*, *VvC4L*, *VvCHS*, *VvCHI*, *VvF3H*, *VvF3’H*, *VvF3’5’H*, *VvDFR*, *VvUFGT,* and *VvLDOX*) peaked at 28DAT, then their expression levels were downregulated (Figure 6B). GA treatment reduced the expression levels of anthocyanin synthesis related genes to some extent, but GA treatment significantly enhanced the expression levels of *VvPAL*, *Vv4CL, VvC4H, VvF3’H, VvUFGT,* and *VvLDOX* in the middle of color conversion (14DAT), which indicates that GA is mainly involved in the formation of berry color in the middle stages of color conversion. ABA and MeJA treatments significantly upregulated the expression of anthocyanin structural genes in the early color-change period (7DAT–14DAT). After exogenous ABA and MeJA treatment, the expression levels of most anthocyanin structural genes reached the maximum at 7DAT and 14DAT, and the expression of anthocyanin structural genes was higher in ABA treatment than in MeJA treatment. Furthermore, we found that NAA treatment generally downregulated the expression level of anthocyanin structural genes and delayed the peak expression of anthocyanin structural genes to 35DAT, indicating that NAA inhibits the expression level of anthocyanin structural genes and is mainly involved in the formation of berry color in the mature stage (Figure 7).

### 3.5. Interaction Mode Variation of VvmiR156b/c/d-VvSPL9 Modules in Response to Hormone Signal in the Regulation of the Grape Color Formation

By comparing the expression levels of VvmiR156b/c/d-*VvSPL9* and anthocyanin synthesis-related genes (Figure S3), we found that during grape berry development, the expression patterns of VvmiR156b/c/d were positively correlated with the expression patterns of anthocyanin synthesis-related genes. Among them, the expression of VvmiR156c/d had a significant positive correlation with the expression of anthocyanin synthesis-related genes including *VvPAL*, *Vv4CL*, *VvCHS*, *VvCHI*, *VvF3’H*, *VvF3’5’H*, *VvDFR*, *VvUFGT*, and *VvOMT* (r = 0.6352~0.9963), while the correlation between VvmiR156b and anthocyanin synthesis-related genes is poor. VvmiR156b was only positively correlated with *VvLDOX* and *VvUFGT* (r = 0.3451~−0.4372), but *VvSPL9* was negatively correlated with the expression of anthocyanin synthesis-related genes including *VvPAL*, *VvF3’H*, *VvF3’5 ‘H*, *VvLDOX*, and *VvUFGT* (r = −0.3352~−0.6396). GA and ABA treatment enhanced the positive correlation between VvmiR156b and *VvPAL*, *VvC4H*, *Vv4CL*, boosted the positive correlation between VvmiR156c/d and *VvC4H*, *VvLDOX*, and weakened the positive correlation between VvmiR156c/d and *VvCHS*, *VvCHI*, *VvDFR*, *VvUFGT*. While GA treatment enhanced the negative correlation between *VvSPL9*, *VvPAL*, *VvCHI*, and *VvUFGT*. In addition, ABA treatment significantly boosted the positive correlation between VvmiR156b, *VvF3’H*, *VvF3’5’H*, *VvLDOX*, and *VvUFGT* (r = 0.8592~0.9423), while MeJA enhanced the positive correlation between VvmiR156b and *VvC4L*, *VvF3’H*, *VvF3’5’H*, and boosted the positive correlation between VvmiR156c/d and *VvDFR*. NAA promoted the positive correlation between VvmiR156b and *VvDFR* and between VvmiR156c/d and *VvLDOX*, *VvUFGT,* while the negative correlation between *VvSPL9* and *VvDFR* was enhanced by NAA treatment. In summary, the correlation analysis of temporal and spatial expression showed that VvmiR156b/c/d can negatively regulate *VvSPL9* expression by responding to different hormone signals, modulated the expression levels of genes related to anthocyanin synthesis, and thereby participated in the formation of grape color.

### 3.6. Functional Verification of Overexpression of VvMIR156b/c/d and VvSPL9 in Tomatoes

**Figure 8:**
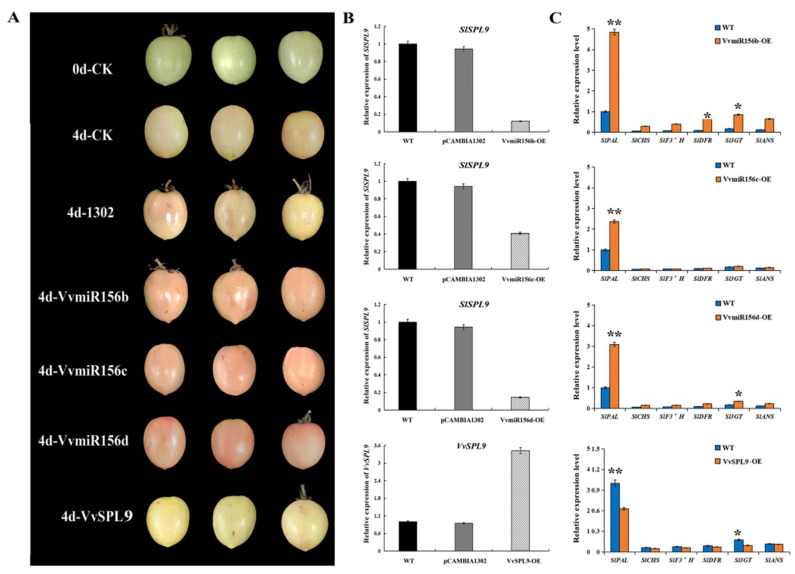
Transient overexpression of VvmiR156b/c/d and *VvSPL9* in tomato. (**A**) VvmiR156b/c/d-OE and*VvSPL9*-OE fruits four days after infiltration. (**B**) The expression of *SPL9* in tomato. (**C**) VvmiR156b/c/d-OE and*VvSPL9*-OE effects on the expression of anthocyanin-related genes in tomato. ANOVA test was used to identify significant differences, asterisks indicate statistically significant differences at (* *p* < 0.05; ** *p* < 0.01) as determined by the Student’s *t*-test.

After four days of fruit injection, the overexpression of tomatoes (OE) was further observed (Figure 8A). Compared with the control plants (CK), VvmiR156b/c/d-OE fruits had a redder color, while *VvSPL9*-OE inhibited tomato coloring. The qRT-PCR results showed that in VvmiR156b/c/d-OE fruits, the expression of *SlSPL9* was inhibited to varying degrees, while in *VvSPL9*-OE fruits, the expression of *VvSPL9* was significantly upregulated (Figure 8B). In addition, we found that in VvmiR156b/c/d-OE fruits, anthocyanin metabolism-related genes were highly expressed; in particular, the expression of *SlPAL*, *SlDFR*, *Sl3GT,* and *SlANS* were significantly upregulated; while in *VvSPL9*-OE fruits, the expression levels of *SlPAL* and *Sl3GT* were inhibited (Figure 8C). These phenomena indicated that VvmiR156b/c/d could promote the expression of anthocyanin synthetic genes by negative regulatory *VvSPL9* expression, which further led to the formation of fruit color.

## 4. Discussion

### 4.1. Regulatory Genes for Anthocyanin Biosynthesis

Anthocyanin belongs to the flavonoid class. Genes involved in anthocyanin biosynthesis are divided into structural and regulatory genes [8,11,33]. Structural genes such as *PAL*, *CHS*, *CHI*, *F3H*, *DFR*, *LDOX,* and *UFGT* directly encode enzymes in anthocyanin biosynthesis pathways and determine the types of anthocyanin synthesis. Regulating genes encoding transcription factors can activate or inhibit the expression patterns and levels of structural genes, thus regulating the strength of anthocyanin synthesis. At present, the regulatory genes of anthocyanin biosynthesis mainly include *R2R3-MYB*, *bHLH,* and *WD40* transcription factors, and *MYB* transcription factors interact with *bHLH* and *WD40* to form the WBM complex to regulate the expression of related structural genes [33,34]. Moreover, transcription factors such as *PIF3*, *HY5*, *COP1*, *WRKY*, *WIP*, *MADS*-box, *NAC*, *SPL* are also involved in the transcriptional regulation of anthocyanin synthesis [35]. Furthermore, miRNAs are involved in the regulation of plant anthocyanin biosynthesis in plants at the post-transcriptional level such as miR858-*MYB111*/*MYB12*, miR828-*MYB75*/*MYB113*, miR827-*NLA,* and miR408-*SPL7*/*HY5* [11,14,22,36]. These provide a new way to study the biosynthesis mechanism of anthocyanin.

It is generally believed that the expression of genes involved in anthocyanin synthesis increases with the maturation of grape and the deepening of pericarp color. In this study, with the ripening and color formation of grape berries, the expression levels of most anthocyanin structural genes reached their peak at 28DAT, then the expression levels were downregulated. VvmiR156b/c/d showed similar expression trends with anthocyanin structural genes, and the expression pattern of VvmiR156b/c/d was positively correlated with anthocyanin contents and anthocyanin synthesis related gene expression, and the expression pattern of *VvSPL9* showed a negative correlation. These findings indicate that VvmiR156b/c/d could promote the expression of anthocyanin synthetic genes by negative regulatory *VvSPL9* expression, thus promoting coloring.

In addition, we constructed the overexpression vectors of VvmiR156 and *VvSPL9* to further verify their functions. However, since grape transgenic technology is not perfect and mature, while tomato is a model transgenic plant system and is thus widely used in the verification of gene functions. Therefore, we utilized transient transformation of injecting tomato fruit for determination of gene functions in this work. Interestingly, four days after injection, all VvmiR156-OE fruits showed a redder color, but *VvSPL9*-OE fruits had inhibited coloring. Meanwhile, in VvmiR156-OE fruits, the expression levels of anthocyanin metabolism-related genes had a higher expression, in particular, *SlPAL*, *SlDFR*, *Sl3GT*, and *SlANS* were significantly upregulated, but the expression of *SlPAL* and *Sl3GT* was inhibited in *VvSPL9*-OE fruits. Similarly, the regulation of anthocyanin synthesis by the miR156-*SPL* pathway also exists in *Arabidopsis thaliana,* apple, litchi, and rice [22,24,25], indicating that the regulation of miR156-*SPL* on plant-specific metabolites is highly conserved in different species, which also provides an idea for the functional study of miR156-*SPL* in other species.

### 4.2. Regulatory Mechanism of Hormones on Anthocyanin Synthesis

Hormone is one of the most important factors affecting anthocyanin synthesis [37,38]. Studies have shown that plant endogenous hormones can affect regulatory genes to control anthocyanin biosynthesis. Meanwhile, the accumulation of anthocyanin can also be controlled by activating or inhibiting the expression of genes related to anthocyanin synthesis through the application of exogenous hormones [39]. Thus far, eight kinds of plant hormones have been found: auxin (IAA), gibberellin (GA), cytokinin (CTK), abscisic acid (ABA), ethylene (ETH), brassinosteroid (BR), jasmonic acid (JA), and salicylic acid (SA). According to its effect, plant hormones can be divided into growth regulators and growth inhibitors [40,41,42]. A large number of studies have shown that ABA has an obvious effect on anthocyanin accumulation in non-climacteric fruits (such as grape, cherry, strawberry, and litchi), while it had no significant effect on climacteric fruits (such as apple) [43,44,45]. Exogenous ABA treatment can induce the expression of structural genes and regulatory genes for anthocyanin in grape skins and cell suspension, and promote the accumulation of anthocyanin [46]. Ethylene plays an important role in the biosynthesis of anthocyanin, 2-chloroethyl phosphonic acid treatment can upregulate anthocyanin synthesis structure genes (*CHS*, *F3H*, *LDOX,* and *UFGT*), and increase the content of anthocyanin [47]. Jasmonic acid (JA) can also induce anthocyanin biosynthesis. In *Arabidopsis thaliana*, JA has synergistic regulatory effects on sucrose-induced *DFR* gene expression and anthocyanin synthesis [48]. JAZ protein can interact with bHLHs (*GL3*, *EGL3*, *TT8*) and R2R3-MYB_S_ (*PAP1*, *PAP2*) transcription factors in the MBW complex to inhibit anthocyanin synthesis [49]. Auxin is also an important hormone that affects anthocyanin synthesis. After NAA treatment, the relative expression levels of anthocyanin synthesis-related structural genes (*CHS*, *CHI*, *F3H*, *F3 ‘5’ H*, *DFR*, *LDOX*, *UFGT*) in grape skins were lower than that in the control group, indicating that exogenous NAA treatment inhibited anthocyanin synthesis [50]. There are few studies on the effect of GA on fruit coloring. DELLA protein (encoded by *GAI* gene) is a negative regulator of GA signal. *gai* mutants do not affect GA synthesis but affect GA signal transduction. The expression level of the *DFR* gene in seedlings and leaves of the *gai* mutant was much higher than that of the wild-type, indicating that GA regulates anthocyanin biosynthesis through the DELLA protein [48].

**Figure 9:**
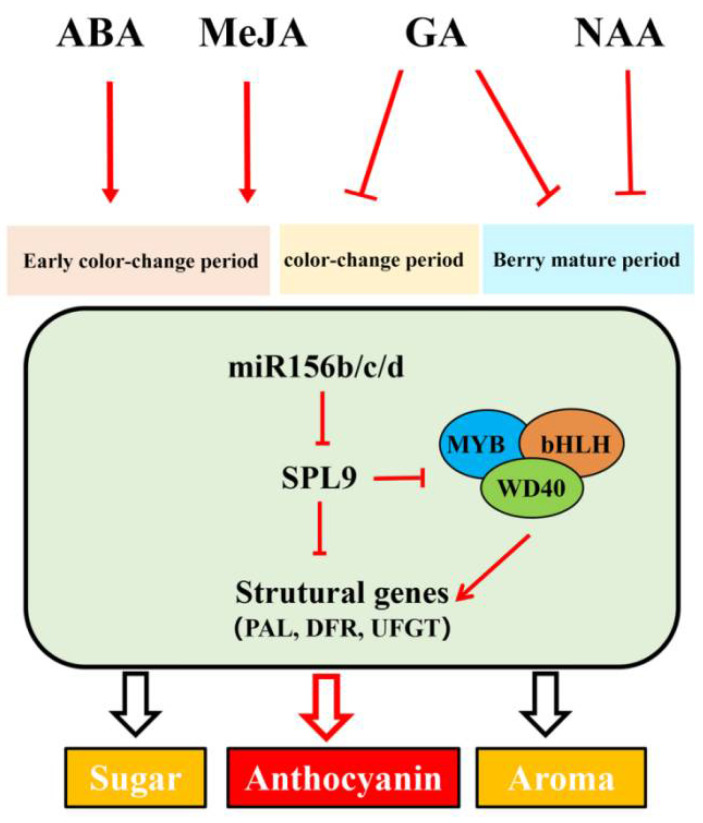
The network of anthocyanins regulated by VvmiR156b/c/d-*VvSPL9* responds to hormones.

Similar to protein-coding genes, the promoter region of plant miRNA genes contains a variety of important cis-acting elements, which are key to the regulation of gene expression. Through promoter action element analysis, we found that the promoter sequence of vmiR156b/c/d contained a large number of hormone-related elements including ABA (ABRE), GA (GARE-motif, P-box, TATC-box), MeJA (CGTCA-motif, TGACG-motif), and auxin (TGA-element) responsive cis-elements. Of these hormone cis-acting elements, ABA and MeJA had the most cis-acting elements, followed by GA, while NAA had the least cis-acting elements. Based on this, ABA, GA, MeJA, and NAA were used to treat grape leaves and fruits, respectively, to verify the short-term and long-term effects of VvmiR156b/c/d and *VvSPL9* response hormones. Interestingly, GA treatment downregulated the expression levels of VvmiR156b/c/d and anthocyanin structural genes in the early color-change period, but significantly upregulated the expression in the middle color-change and ripening stages. In addition, GA upregulated the expression levels of anthocyanin structural genes in the middle color-change stage and ripening stage, so GA mainly promoted grape berry coloring in the middle- and late-ripening stages. After ABA and MeJA treatment, grape berries can quickly color. ABA and MeJA significantly upregulated the expression levels of VvmiR156b/c/d and anthocyanin structural genes in the early stage of color conversion (7DAT and 14DAT), and ABA and MeJA are mainly involved in the formation of color in the early color-change period. NAA treatment downregulated the expression of VvmiR156b/c/d and anthocyanin structural genes; NAA delayed the peak expression of genes to 35DAT and is mainly involved in the formation of color in the mature stage. Thus, we conclude that VvmiR156b/c/d-*VvSPL9* can respond to different hormone signals to participate in the formation of anthocyanin of different periods (Figure 9), but the mechanism of the hormone signaling pathway remains unclear. At present, a variety of miRNA has been proved to be involved in the response and synthesis of plant hormones. miR167 affects the development of pistils, stamens, and petals of Arabidopsis thaliana by regulating the expression of ARF6 and ARF8 [51], and miRl60 affects the formation of root cap cells by regulating the expression of ARF10 and ARF16 [52]. DELLA, the key molecule of gibberellin signal transmission, has a direct protein interaction with the target gene SPL of miR156. Gibberellin is regulated by the transcription factor of the target gene SPL of miR156 [53]. More interestingly, miR159 appears in many plant hormones signaling pathways. It can be up-regulated by the regulation of GA and ABA [54,55], and it will be down-regulated by the regulation of ethylene [56]. There are various kinds of miRNAs, and with the discovery of new miRNAs in different species, there is still a long way to go to clarify the relationship between miRNAs and plant hormones.

## Figures and Tables

**Table 1:** Physiological indexes of grape berry treated with different hormones.

Physiological Indexes	Different Treatment Periods					
	Treatment	7DAT	14DAT	21DAT	28DAT	35DAT
transverse diameter (mm)	CK	20.53 ± 0.59 ^b^	21.35 ± 0.61 ^bc^	20.57 ± 0.58 ^b^	21.75 ± 0.73 ^b^	21.54 ± 0.79 ^b^
	GA	19.87 ± 0.63 ^c^	21.19 ± 0.74 ^c^	20.34 ± 0.36 ^b^	20.98 ± 0.63 ^c^	21.31 ± 0.82 ^b^
	ABA	21.01 ± 0.34 ^a^	21.96 ± 0.58 ^a^	21.54 ± 0.41 ^a^	22.12 ± 0.25 ^b^	22.27 ± 0.29 ^a^
	MeJA	20.94 ± 0.64 ^a^	21.73 ± 0.62 ^ab^	21.23 ± 0.76 ^a^	22.67 ± 0.59 ^a^	22.16 ± 0.31 ^a^
	NAA	19.28 ± 0.58 ^d^	19.43 ± 0.54 ^d^	19.31 ± 0.49 ^c^	19.84 ± 0.62 ^d^	20.06 ± 0.76 ^c^
longitudinal diameter (mm)	CK	28.12 ± 0.62 ^b^	28.91 ± 0.77 ^b^	29.14 ± 0.59 ^ab^	29.11 ± 0.99 ^ab^	29.42 ± 0.89 ^bc^
	GA	27.36 ± 0.86 ^c^	28.20 ± 0.94 ^c^	28.85 ± 0.63 ^b^	28.82 ± 0.83 ^b^	29.17 ± 0.68 ^c^
	ABA	28.58 ± 0.74 ^a^	29.31 ± 0.96 ^a^	29.45 ± 0.74 ^a^	29.50 ± 0.39 ^a^	29.90 ± 0.43 ^a^
	MeJA	28.49 ± 0.57 ^ab^	29.15 ± 0.62 ^ab^	29.32 ± 0.92 ^a^	29.31 ± 0.65 ^a^	29.61 ± 1.03 ^ab^
	NAA	26.82 ± 0.94 ^d^	27.31 ± 0.54 ^d^	27.89 ± 0.91 ^c^	28.33 ± 0.78 ^c^	28.62 ± 0.73 ^d^
berry weigh (g)	CK	6.13 ± 0.16 ^a^	6.81 ± 0.14 ^bc^	8.06 ± 0.09 ^ab^	8.31 ± 0.15 ^a^	8.53 ± 0.14 ^b^
	GA	6.05 ± 0.16 ^a^	6.56 ± 0.22 ^c^	7.78 ± 0.35 ^b^	8.23 ± 0.11 ^ab^	8.38 ± 0.19 ^b^
	ABA	6.23 ± 0.19 ^a^	7.72 ± 0.21 ^a^	8.39 ± 0.38 ^a^	8.50 ± 0.22 ^a^	8.92 ± 0.29 ^a^
	MeJA	6.19 ± 0.12 ^a^	6.97 ± 0.23 ^b^	8.18 ± 0.23 ^a^	8.41 ± 0.27 ^a^	8.61 ± 0.26 ^ab^
	NAA	6.10 ± 0.18 ^a^	6.57 ± 0.15 ^c^	7.32 ± 0.17 ^c^	7.84 ± 0.14 ^b^	7.97 ± 0.13 ^c^
soluble solid (%)	CK	12.57 ± 0.22 ^c^	15.61 ± 0.21 ^b^	17.05 ± 0.39 ^c^	18.12 ± 0.47 ^c^	21.21 ± 0.73 ^c^
	GA	12.25 ± 0.37 ^c^	12.57 ± 0.22 ^c^	15.41 ± 0.21 ^d^	17.58 ± 0.39 ^d^	19.09 ± 0.61 ^d^
	ABA	14.63 ± 0.41 ^a^	16.46 ± 0.52 ^a^	18.81 ± 0.52 ^a^	20.13 ± 0.52 ^a^	22.71 ± 0.67 ^a^
	MeJA	13.84 ± 0.52 ^b^	15.89 ± 0.34 ^b^	17.93 ± 0.62 ^b^	19.44 ± 0.69 ^b^	21.82 ± 0.59 ^b^
	NAA	11.39 ± 0.23 ^d^	12.46 ± 0.31 ^c^	14.82 ± 0.39 ^e^	16.37 ± 0.43 ^e^	17.54 ± 0.41 ^e^

Note: data are means ± standard error among three biological replicates, different letters indicate the significance of the different treatments is at the level of *p* < 0.05, the same below.

## Data Availability

Data is contained within the article or Supplementary Materials.

## Supplementary Materials

The following are available online at https://www.mdpi.com/article/10.3390/foods10040896/s1, Table S1: The cloning primers of VvmiR156b/c/d and *VvSPL9*; Table S2: The cloning primers of VvmiR156b/c/d and *VvSPL9* promoters; Table S3: Quantitative primers of VvmiR156b/c/d and *VvSPL9*; Table S4: Quantitative primers for anthocyanin synthesis genes of grape; Table S5: Quantitative primers for anthocyanin synthesis genes of tomato; Table S6: Volatile substances in ‘Wink’; Table S7: Classification and functional analysis of the promoter of VvmiR156b/c/d; Figure S1: HPLC chromatogram of anthocyanins standard sample (cyanidin-3-O-glucoside chloride); Figure S2: GC-MS chromatogram of ‘Wink’ berry flesh; Figure S3: The correlation analysis of VvmiR156b/c/d-*VvSPL9* and anthocyanin synthesis-related genes in response to hormone signals.
