# Supplementary material for: Characterization and Action Mechanism Analysis of VvmiR156b/c/d-VvSPL9 Module Responding to Multiple-Hormone Signals in the Modulation of Grape Berry Color Formation
Source: Foods. 2021 Apr 19;10(4):896. doi: 10.3390/foods10040896 (PMC8073990; doi:10.3390/foods10040896)
Supplement: Supplementary file 1 [file foods-10-00896-s001.zip › foods-1133282-supplementary.pdf]

Supplementary Material

Table S1. The cloning primers of VvmiR156b/c/d and *VvSPL9*.

Primer name	Forward primer sequences (5'-3')	Reverse primer sequences (5'-3')	Use
VvmiR156b	CATGCCATGGGTAGCAGAGAGAGACCGG AGCTT	GACTAGTCTCTCTTTTCATTTTAAAGTCGCTCT	VvmiR156b clone
VvmiR156c	CATGCCATGGCACTTGAGAGATGAGACT CAAACTG	GACTAGTCTCACATTTATTCCAATGTCCA	VvmiR156c clone
VvmiR156d	CATGCCATGGCCGGAGAGGGAGGGGAAC TGAAC	GACTAGTAGTGTGAGGCTTTGGGGGGATTATG	VvmiR156d clone
VvSPL9	ACACGGGGGACTCTTGACCATGGTAGAT CTATGGAAAGGGGTTCGAGCT	TCCAGTGAAAAGTTCTTCTCCTTTACTAGTCT AAAGTGACCAGTGCATCTGCT	VvSPL9 clone

Table S2 The cloning primers of VvmiR156b/c/d and *VvSPL9* promoters.

Primer name	Primer sequence	Primer use
P-VvmiR156b-F	CAGCTATGACCATGATTACGCCAAGCTTCTGGATTTGACACAAGATTGGA	Amplification of
P-VvmiR156b-R	ACATAAGGGACTGACCACCCGGGGATCCTGCCCCAGAAGAAGAAGAAAG	VvmiR156b promoter
P-VvmiR156c-F	CAGCTATGACCATGATTACGCCAAGCTTACTCCCCTCATCTCCCACTCT	Amplification of
P-VvmiR156c-R	ACATAAGGGACTGACCACCCGGGGATCCACCATTTGTAACTCCCATACAAACA	VvmiR156c promoter
P-VvmiR156d-F	CAGCTATGACCATGATTACGCCAAGCTTGACAATGATACGGCCATTGA	Amplification of
P-VvmiR156d-R	ACATAAGGGACTGACCACCCGGGGATCCCGTCTCCCTCTTATCTTCGC	VvmiR156d promoter

Table S3 Quantitative primers of VvmiR156b/c/d and *VvSPL9*.

Primer name	Primer sequence	Primer use
qVvmiR156b-F	TTGGACTTCAACACCCAACA	qRT-PCR for VvmiR156b
qVvmiR156b-R	TTCTTCTTCTGGGGCACATT	
qVvmiR156c-F	GCAGCTCCTTCTGTTCCATC	qRT-PCR for VvmiR156c
qVvmiR156c-R	CATGGTCTGTTCTTGCATGG	
qVvmiR156d-F	GAGAGGGAGGGGAACTGAAC	qRT-PCR for VvmiR156d
qVvmiR156d-R	CTCATCCCTTCTGCTTCTGC	
qVvSPL9-F	TTCGAGCTCTTTGACCGTTT	qRT-PCR for VvSPL9
qVvSPL9-R	CTCCCTGAACAACCCCACTA	

Table S4 Quantitative primers for anthocyanin synthesis genes of grape.

Primer name	Primer sequence	Primer use
qVvPAL-F	CATCGAACGGGAGATCAACT	qRT-PCR for VvPAL
qVvPAL-R	TGATGGCAGTCCATTGTTGT	
qVv4CL-F	ACCACCTCCCTCTCCACAC	qRT-PCR for Vv4CL
qVv4CL-R	GCTCCGAGAAAGGAGAACG	
qVvC4H-F	CGTTGTTCGTGAAGCTCAAA	qRT-PCR for VvC4H
qVvC4H-R	GTCCTTGAAGAGCTGCAACC	
qVvCHS-F	GAAGATGGGAATGGCTGCTG	qRT-PCR for VvCHS
qVvCHS-R	AAGGCACAGGGACACAAAAG	
qVvCHI-F	TCCAGATCAAGTTCACAGCA	qRT-PCR for VvCHI
qVvCHI-R	GAAATAAGAGCCTCAAAGAA	
qVvF3H-F	CTGTGGTGAACTCCCGACTGC	qRT-PCR for VvF3H
qVvF3H-R	CAAATGTTATGGGCTCCTCC	

qVvF3'H-F	GCCTCCGTTGCTGCTCAGTT	qRT-PCR for VvF3'H
qVvF3'H-R	GAGAAGAGGTGGACGGAGCAAATC	
qVvF3'5'H-F	AAACCGCTCAGACCAAAACC	qRT-PCR for VvF3'5'H
qVvF3'5'H-R	ACTAAGCCACAGGAAACTAA	
qVvDFR-F	GAAACCTGTAGATGGCAGGA	qRT-PCR for VvDFR
qVvDFR-R	GGCCAAATCAAACTACCAGA	
qVvLDOX-F	AGGGAAGGGAAAACAAGTAG	qRT-PCR for VvLDOX
qVvLDOX-R	ACTCTTTGGGGATTGACTGG	
qVvOMT-F	CCAAGGGTGTCGTCCATATC	qRT-PCR for VvOMT
qVvOMT-R	GGAATTGAACTCGGCAGAAG	
qVvUFGT-F	GGGATGGTAATGGCTGTGG	qRT-PCR for VvUFGT
qVvUFGT-R	ACATGGGTGGAGAGTGAGTT	
qVvActin-F	TACAATTCCATCATGAAGTGTGATG	Actin internal reference primer
qVvActin-R	TTAGAAGCACTTCCTGTGAACAATG	

Table S5 Quantitative primers for anthocyanin synthesis genes of tomato.

Primer name	Primer sequence	Primer use
qSly-CAC-F	CCTCCGTTGTGATGTAACTG	qRT-PCR for Sly-CAC
qSly-CAC-R	ATTGGTGGAAAGTAACATCATC	
qSly-PAL-F	ATTGGGAAATGGCTGCTGATT	qRT-PCR for Sly-PAL
qSly-PAL-R	TCAACATTTGCAATGGATGCA	
qSly-CHS-F	TGGTCACCGTGGAGGAGTATC	qRT-PCR for Sly-CHS
qSly-CHS-R	GATCGTAGCTGGACCCTCTGC	
qSly-F3'H-F	GCACCACGAATGCACTTGC	qRT-PCR for Sly-F3'H
qSly-F3'H-R	CGTTAGTACCGTCGGCGAAT	
qSly-DFR-F	CAAGGCAGAGGGAAGATTCATTTG	qRT-PCR for Sly-DFR
qSly-DFR-R	GCACCATCTTAGCCACATCGTA	
qSly-3GT-F	GCACATAAGAGTGTTGGCGTTT	qRT-PCR for Sly-3GT
qSly-3GT-R	TTTCCAAACACTTTCCACCA	
qSly-ANS-F	GAACTAGCACTTGGCGTCGAA	qRT-PCR for Sly-ANS
qSly-ANS-R	TTGCAAGCCAGGCACCATA	
qSly-SPL9-F	GGTGCCAAGTTGAAGGTTGT	qRT-PCR for Sly-SPL9
qSly-SPL9-R	CCCCTGGTCGAATTCAGTTA	

Table S6 Volatile substances in 'Wink'.

Component Name	Retention Time	Reference m/z	BP Area	Content (mg)	Content ratio	BP Height	TIC	Formula (mol ion)	CAS No.	SI	RSI
2-Hexenal	5.752	41.039	125997816	1.06046E-06	9.97%	34209494	212616618	C6H10O	505-57-7	957	958
3-Hexen-1-ol, (Z)-	5.899	41.039	88262562	7.42863E-07	6.98%	15684067	54390486	C6H12O	928-96-1	830	835
Formamide, N,N-dibutyl-	28.15	71.98	84637002	7.12348E-07	6.70%	17091297	48404241	C9H19NO	761-65-9	834	834
Benzaldehyde, 2,4-dimethyl-	23.818	133.062	76969388	6.47814E-07	6.09%	13955967	69529075	C9H10O	15764-16-6	932	932
Toluene	3.488	91.062	74335451	6.25645E-07	5.88%	20112981	49714422	C7H8	108-88-3	929	929
2-Buten-1-one, 1-(2,6,6-trimethyl-1,3-cyclohexadien-1-yl)-, (E)-	32.148	69.088	63181465	5.31767E-07	5.00%	16062904	63290033	C13H18O	23726-93-4	914	944
2-Heptenal, (Z)-	10.531	41.039	63162446	5.31607E-07	5.00%	12171274	77558899	C7H12O	57266-86-1	937	947
5,9-Undecadien-2-one, 6,10-dimethyl-, (E)-	34.502	43.03	62407156	5.2525E-07	4.94%	21715195	78471942	C13H22O	3796-70-1	897	897
2-Octenal, (E)-	15.91	41.039	51865288	4.36525E-07	4.10%	11578850	82449267	C8H14O	2548-87-0	869	869
1-Octen-3-one	11.738	55.03	51817744	4.36125E-07	4.10%	8997180	28841507	C8H14O	4312-99-6	873	883
Trichloromethane	1.925	83.055	40415625	3.40159E-07	3.20%	19332983	50728379	CHCl3	67-66-3	940	940
Hexanal	4.136	41.039	39465304	3.3216E-07	3.12%	12394274	69551462	C6H12O	66-25-1	886	886
Nonanal	18.385	57.063	34840153	2.93232E-07	2.76%	8883263	75489308	C9H18O	124-19-6	938	938
**Octanal**	13.123	41.039	29406488	2.475E-07	2.33%	4121327	23434284	C8H16O	124-13-0	835	835
3-Buten-2-one, 3-methyl-	2.288	41.039	27577779	2.32109E-07	2.18%	10135478	40840268	C5H8O	814-78-8	912	912
n-Propyl acetate	2.683	43.03	25736155	2.16609E-07	2.04%	8704407	16157322	C5H10O2	109-60-4	936	938
Cyclopropane, propyl-	6.463	56.077	25321726	2.13121E-07	2.00%	4538826	14337615	C6H12	2415-72-7	892	899
2,6-Octadiene, 2,6-dimethyl-	15.699	41.039	24077891	2.02652E-07	1.91%	5656647	24250016	C10H18	2792-39-4	825	843
Pentanal	2.492	44.046	22267519	1.87415E-07	1.76%	8190054	26787576	C5H10O	110-62-3	870	895

Tetradecane	32.839	57.063	20201072	1.70023E-07	1.60%	5748057	28037470	C14H30	629-59-4	890	891
1-Propanone, 1-cyclopropyl-	3.539	41.039	18395435	1.54825E-07	1.46%	5199460	15067304	C6H10O	6704-19-4	863	877
1-Ethylhexyl propionate	23.781	57.063	18363939	1.5456E-07	1.45%	5156511	11029909	C11H22O2		874	899
2-Propyl-1-pentanol	14.505	57.063	17647825	1.48533E-07	1.40%	3642643	12675733	C8H18O	58175-57-8	896	898
Ethyl Acetate	1.905	43.03	12016625	1.01138E-07	0.95%	3948904	7062476	C4H8O2	141-78-6	878	878
Decanal	23.553	41.039	11135666	9.37234E-08	0.88%	2498923	24862351	C10H20O	112-31-2	914	914
7-Oxabicyclo[4.1.0]heptane	5.879	83.055	8701310	7.32347E-08	0.69%	2070684	3390487	C6H10O	286-20-4	911	911
2,4-Di-tert-butylphenol	35.978	191.142	8683838	7.30876E-08	0.69%	3410510	11749884	C14H22O	96-76-4	917	918
Phenylethyl Alcohol	18.791	91.062	7768862	6.53867E-08	0.61%	795361	1884354	C8H10O	22258	912	912
p-Xylene	7.462	91.062	6413030	5.39753E-08	0.51%	1022983	1794916	C8H10	106-42-3	862	862
Cyclopropane, pentyl-	16.721	41.039	5241120	4.41119E-08	0.41%	1209223	7604331	C8H16	2511-91-3	847	859
Pentadecane	35.686	57.063	4952291	4.1681E-08	0.39%	1902668	9610062	C15H32	629-62-9	900	901
Diethyltoluamide	37.312	119.03	4940193	4.15792E-08	0.39%	1590855	4105828	C12H17NO	134-62-3	920	922
trans-2-Nonenal	21.219	41.039	4875490	4.10346E-08	0.39%	1063465	8757090	C9H16O		920	922
2-Undecenal	31.142	41.039	4552501	3.83162E-08	0.36%	811170	6863181	C11H20O	2463-77-6	868	869
2-Decenal, (Z)-	26.249	41.039	4473326	3.76498E-08	0.35%	991011	9331213	C10H18O	2497-25-8	922	926
2(3H)-Furanone, 5-acetyldihydro-	15.668	84.946	4296923	3.61651E-08	0.34%	1275887	3238289	C6H8O3	29393-32-6	815	878
Benzeneacetaldehyde	15.172	91.062	3801711	3.19971E-08	0.30%	404950	740491	C8H8O	122-78-1	865	892
Cyclohexanol, 5-methyl-2-(1-methylethyl)-, (1.alpha.,2.beta.,5.alpha.)-(図)-	21.826	71.093	3800843	3.19898E-08	0.30%	628145	4828509	C10H20O	15356-70-4	885	885
Linalool	18.177	71.093	3554353	2.99152E-08	0.28%	739385	5440785	C10H18O	78-70-6	858	858
Benzaldehyde	10.722	77.037	3538019	2.97778E-08	0.28%	294101	807286	C7H6O	100-52-7	837	837
1-Butanol, 3-methyl-	2.938	55.03	3448746	2.90264E-08	0.27%	1138350	5811306	C5H12O	123-51-3	912	912
Tridecane	23.318	57.063	3396541	2.8587E-08	0.27%	742599	3388291	C13H28	629-50-5	835	854
Ethylbenzene	6.141	91.062	3351464	2.82076E-08	0.27%	626252	1266307	C8H10	100-41-4	868	876
Heptanal	7.902	41.039	3289777	2.76884E-08	0.26%	704199	4462370	C7H14O	111-71-7	852	852
2-Propenoic acid, oxiranylmethyl ester	2.412	55.03	3223534	2.71309E-08	0.26%	1064045	1231080	C6H8O3	106-90-1	874	874
3-Tetradecene, (Z)-	32.524	55.03	3122475	2.62803E-08	0.25%	699388	5193984	C14H28	41446-67-7	890	893
Nitrogen fluoride, (E)-	12.734	66.073	3099237	2.60848E-08	0.25%	712617	827011	F2N2	13776-62-0	856	999
3-Tetradecene, (Z)-	22.889	41.039	3019835	2.54165E-08	0.24%	735828	6597666	C14H28	41446-67-7	927	928
Pyrimidine-2,4(1H,3H)-dione, 5-amino-6-nitroso-	1.811	43.03	2765388	2.32749E-08	0.22%	553550	911199	C4H4N4O3		823	902
Phthalic acid, hex-3-yl isobutyl ester	41.404	149.015	2563642	2.15769E-08	0.20%	1009310	2027685	C18H26O4		901	904
Dibutyl phthalate	42.467	149.015	2466218	2.07569E-08	0.20%	940159	1880187	C16H22O4	84-74-2	897	907
Propanoic acid, 2-methyl-, 3-hydroxy-2,2,4-trimethylpentyl ester	31.585	71.093	2361813	1.98782E-08	0.19%	374859	1339054	C12H24O3	77-68-9	806	811
Undecane, 4,7-dimethyl-	27.188	71.093	2324283	1.95624E-08	0.18%	535098	2329513	C13H28	17301-32-5	883	885
1-Pentanol	3.482	41.039	2305126	1.94011E-08	0.18%	1039979	4177584	C5H12O	71-41-0	827	844
3-Buten-2-one, 4-(2,6,6-trimethyl-1-cyclohexen-1-yl)-	35.374	177.132	2154594	1.81342E-08	0.17%	757255	3678963	C13H20O	14901-07-6	865	865
Hexane, 2,2,5-trimethyl-	26.846	71.093	2027181	1.70618E-08	0.16%	273831	367046	C9H20	3522-94-9	937	942
1,2,4-Triazin-5(4H)-one, 3,4-diamino-6-methyl-	10.571	141.06	1892690	1.59298E-08	0.15%	380924	918562	C4H7N5O	52553-11-4	845	845
Pentane, 1-chloro-	3.264	42.032	1843712	1.55176E-08	0.15%	570967	2822340	C5H11Cl	543-59-9	899	899
Hexane, 2,2,5-trimethyl-	31.185	71.093	1789429	1.50607E-08	0.14%	263507	386526	C9H20	3522-94-9	816	940
Cyclopropane, 1,1'-methylenebis-	31.953	68.115	1739926	1.46441E-08	0.14%	278101	342009	C7H12	5685-47-2	856	874
Hexadecanoic acid, ethyl ester	42.715	88.065	1725762	1.45249E-08	0.14%	682582	3320591	C18H36O2	628-97-7	834	834
3-Hydroxy-3-phenylbutan-2-one	17.372	43.03	1547419	1.30239E-08	0.12%	325037	704456	C10H12O2	458389	814	830
Sulphuric acid dibutyl ester	2.992	41.039	1526116	1.28446E-08	0.12%	468373	1015003	C8H18O4S	625-22-9	801	804
Decane, 2,3,5,8-tetramethyl-	34.77	57.063	1422240	1.19703E-08	0.11%	434794	2117472	C14H30	192823-15-7	828	830
Propanedioic acid, propyl-	22.081	60.073	1410373	1.18704E-08	0.11%	244771	715534	C6H10O4	616-62-6	868	874
Naphthalene, 1,2,3,4-tetrahydro-1,1,6-trimethyl-	23.707	159.118	1362600	1.14683E-08	0.11%	279973	750748	C13H18	475-03-6	848	852
Sulfurous acid, 2-ethylhexyl hexyl ester	35.602	57.063	1347453	1.13409E-08	0.11%	538269	3174168	C14H30O3S		820	845
Cyclopropane, nonyl-	35.069	55.03	1194111	1.00502E-08	0.09%	404726	3721268	C12H24	74663-85-7	847	852
Heptadecane, 2,6-dimethyl-	39.177	57.063	1141476	9.60724E-09	0.09%	503149	2369372	C19H40	54105-67-8	819	822

Methacrolein	1.731	38.991	1131150	9.52034E-09	0.09%	494044	1447273	C4H6O	78-85-3	874	874
Propene	12.107	41.039	1111312	9.35337E-09	0.09%	436401	1013881	C3H6	115-07-1	807	899
N-Benzyloxy-2-carbomethoxyaziridine	15.397	91.062	1018944	8.57595E-09	0.08%	189573	261956	C11H13NO3	53084-33-6	838	856
Diphenylamine	38.057	169.113	980888	8.25565E-09	0.08%	280960	986396	C12H11N	122-39-4	900	915
Octane, 2,2,6-trimethyl-	36.558	71.093	843181	7.09664E-09	0.07%	293069	689284	C11H24	62016-28-8	818	839
2-(1H-Imidazol-1-yl)acetohydrazide	14.636	82.132	821457	6.9138E-09	0.06%	171321	299978	C5H8N4O	56563-00-9	826	876
Heptane, 2,2-dimethyl-	27.852	71.093	754738	6.35226E-09	0.06%	145861	284595	C9H20	1071-26-7	818	824
1H-Indene, 1-methylene-	22.201	128.076	695946	5.85744E-09	0.06%	211990	323041	C10H8	2471-84-3	940	959
3,4-Diamino-1,2,4(4H)-triazole	25.424	43.03	538274	4.53039E-09	0.04%	155606	248808	C2H5N5	38104-45-9	820	852
Benzophenone	38.191	105.072	502884	4.23253E-09	0.04%	122103	437218	C13H10O	119-61-9	799	823
Methane, diethoxy-	2.298	59.031	485925	4.08979E-09	0.04%	211914	363137	C5H12O2	462-95-3	937	937
Cyclotrisiloxane, hexamethyl-	4.766	207.04	422545	3.55635E-09	0.03%	131233	200185	C6H18O3Si3	541-05-9	839	839
3-Octadecene, (E)-	37.5	57.063	340052	2.86205E-09	0.03%	130683	1258199	C18H36	7206-19-1	880	880
Methylene chloride	1.617	48.982	299802	2.52329E-09	0.02%	133507	356185	CH2Cl2	27639	888	888

Table S7 Classification and functional analysis of promoter of VvmiR156b/c/d.

VvmiR156b	SUM	NUM	ELEMENTS	FUCTIONS
Light-related elements	13	2	Box 4	part of a conserved DNA module involved in light responsiveness
		1	G-Box	cis-acting regulatory element involved in light responsiveness
		3	G-box	cis-acting regulatory element involved in light responsiveness
		2	GT1-motif	light responsive element
		2	Sp1	light responsive element
		3	TCT-motif	part of a light responsive element
Hormone-related elements	11	5	ABRE	cis-acting element involved in the abscisic acid responsiveness
		1	CGTCA-motif1	cis-acting regulatory element involved in the MeJA-responsiveness
		1	GARE-motif	gibberellin-responsive element
		1	P-box	gibberellin-responsive element
		1	TATC-box	cis-acting element involved in gibberellin-responsiveness
		1	TCA-element	cis-acting element involved in salicylic acid responsiveness
		1	TGACG-motif	cis-acting regulatory element involved in the MeJA-responsiveness
Stress-related elements	1	1	LTR	cis-acting element involved in low-temperature responsiveness
Circadian-related elements	1	1	circadian	cis-acting regulatory element involved in circadian control

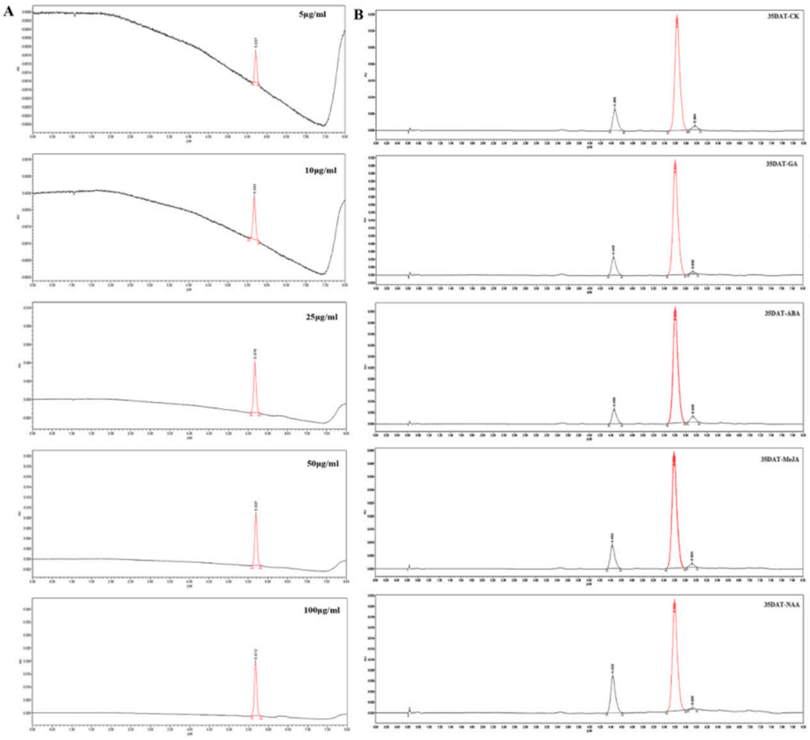

Figure S1 HPLC chromatogram of anthocyanins standard sample (Cyanidin-3-O-glucoside chloride).

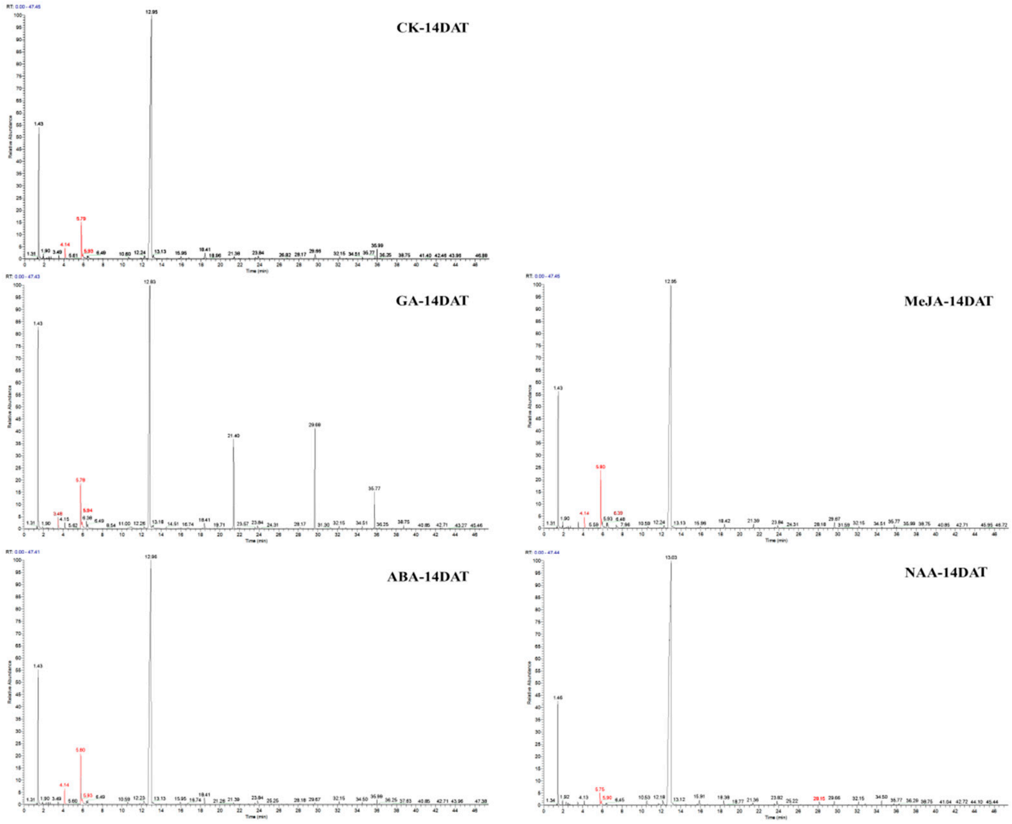

Figure S2 GC–MS chromatogram of 'Wink' berry flesh

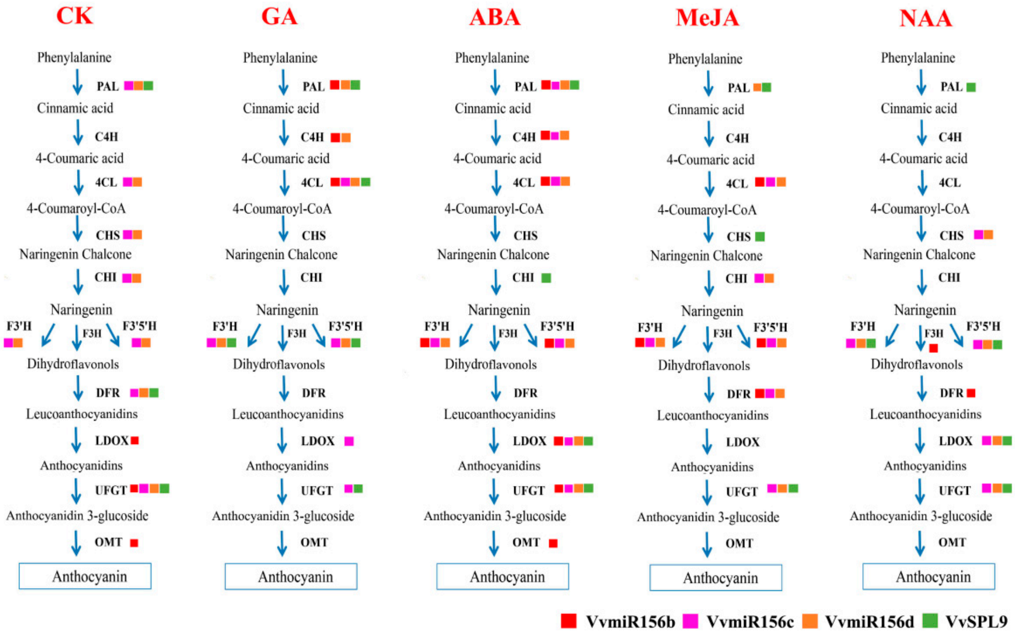

Figure S3 The correlation analysis of VvmiR156b/c/d-*VvSPL9* and anthocyanin synthesis-related genes in response to hormone signal. The red, purple and orange boxes represent the positive correlation of VvmiR156b/c/d, and the green box represents the negative correlation of *VvSPL9*. The larger the box, the stronger the correlation.
